# Flow microreactor synthesis in organo-fluorine chemistry

**DOI:** 10.3762/bjoc.9.314

**Published:** 2013-12-05

**Authors:** Hideki Amii, Aiichiro Nagaki, Jun-ichi Yoshida

**Affiliations:** 1 Division of Molecular Science, Graduate School of Science and Technology, Gunma University, Tenjin-cho, Kiryu, Gunma 376-8515, Japan; 2 Department of Synthetic and Biological Chemistry, Graduate School of Engineering, Kyoto University, Nishikyo-ku, Kyoto 615-8510, Japan

**Keywords:** defluorination, fluorine, fluorination, flow microreactor, organo-fluorine, perfluoroalkylation

## Abstract

Organo-fluorine compounds are the substances of considerable interest in various industrial fields due to their unique physical and chemical properties. Despite increased demand in wide fields of science, synthesis of fluoro-organic compounds is still often faced with problems such as the difficulties in handling of fluorinating reagents and in controlling of chemical reactions. Recently, flow microreactor synthesis has emerged as a new methodology for producing chemical substances with high efficiency. This review outlines the successful examples of synthesis and reactions of fluorine-containing molecules by the use of flow microreactor systems to overcome long-standing problems in fluorine chemistry.

## Review

Fluorine is a key element in the development of materials and biologically active agents. The introduction of fluorine atoms into organic molecules often exerts influences upon physical, chemical, and biological properties [1–8]. Although the fluorine atom is larger than the hydrogen atom, the replacement of hydrogen by fluorine leads to the smallest steric perturbation available to us. In pharmaceuticals, fluorine is often introduced to increase lipophilicity, bioavailability, and metabolic stability; these unique properties are otherwise difficult to obtain. At present, nearly 15% of medicines and 20% of agrochemicals on the market contain fluorine atoms [6,9–10].

Despite increased demands in wide fields of science, synthesis of fluoro-organic compounds is still often faced with problems such as the difficulty in handling of fluorinating reagents and in controlling of chemical reactions. Furthermore, low stability of fluorine-containing intermediates and low selectivity (chemo-, regio-, and/or stereo-) of the reactions have disturbed the progress of synthesis of fluorochemicals. New methodologies that can cure the weak points in synthesizing fluoro-organic compounds would open up a new perspective for fluorine chemistry.

Meanwhile, flow microreactor synthesis, the use of microfluidic devices, has emerged as a new method for producing chemical substances with high efficiency [11–13]. Now, the introduction of continuous-flow synthesis technique to laboratory synthesis represents a highly useful and increasingly popular method in organic chemistry [14–17]. Flow microreactor systems serve as an effective method for precise control of chemical reactions. Not only operational safety, but also extremely fast mixing, efficient heat transfer, and precise residence time control by virtue of the characteristic features of microstructures are responsible for their effectiveness. In particular, brilliant works of the generation and reactions of reactive intermediates that cannot be done in batch reactors have been developed by means of ‘space integration methodology’, where a sequence of reactions is conducted in one flow by adding the reagents at different places in continuous-flow synthesis [18–25].

This review deals with the successful examples on synthesis and reactions of fluorine-containing molecules by the use of flow microreactor systems. Useful applications using flow microreactor technology to overcome long-standing problems in synthetic organofluorine chemistry are showcased.

### Reactions using hazardous fluorinating reagents

**Scheme 1:**
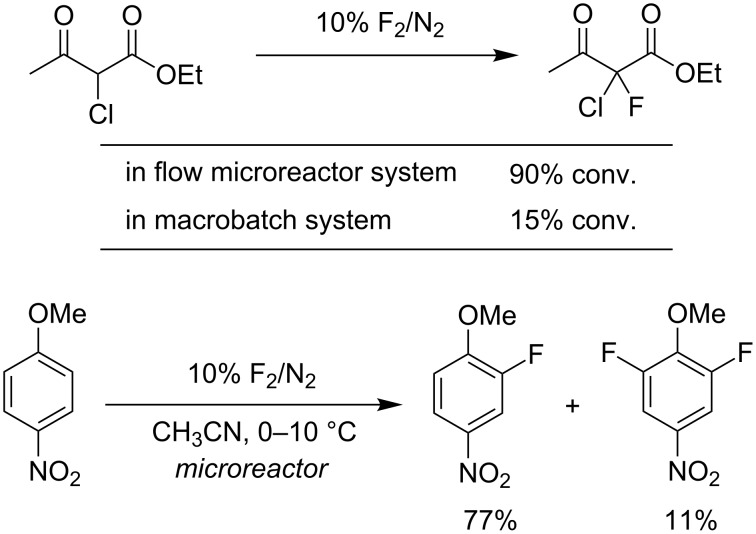
Direct fluorination using microreactor systems.

Direct fluorination employing elemental fluorine (F_2_) is one of the most straightforward methods to make fluorine-containing molecules with high atom economy. However, handling F_2_ needs special care and there are a lot of practical difficulties associated with direct fluorination. Elemental fluorine is a poisonous pale yellow gas at room temperature (bp −188 °C). Reactions of organic substances with elemental fluorine are typically strongly exothermic and extremely fast, frequently explosive. So it is difficult to control direct fluorination reactions with F_2_ gas in conventional batch reactors. In 1999, the pioneering work using flow microreactor technology for direct fluorination was reported by Chambers and Spink (Scheme 1) [26]. Utilizing the flow microreactor system, selective fluorination reactions were accomplished. For instance, fluorination of β-dicarbonyl compounds proceeds with high efficiency, whereas the use of conventional macrobatch systems suffered from low conversion [27–28]. Since then, direct fluorination processes using flow microreactor systems have been studied extensively [29–36]. The reactions of arenes with F_2_ gas cause not only substitution (aromatic fluorination) but also side reactions such as fluorine-addition, polymerization and so on due to the high reactivity of F_2_ gas [37–40]. Direct fluorination of aromatics in microreactor system took place cleanly [36].

**Scheme 2:**
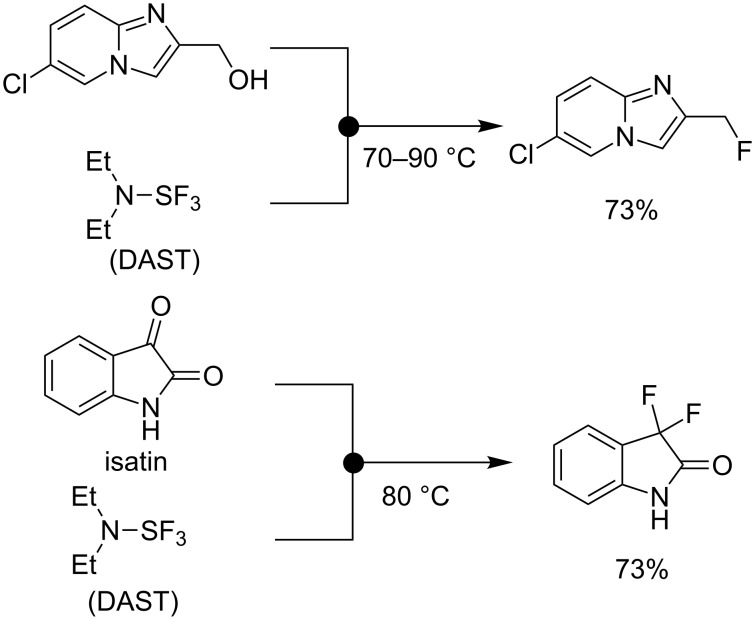
Use of DAST in continuous-flow reactors.

As another splendid example for safe handling of hazardous fluorinating reagents, the flow microreactor synthesis using diethylaminosulfur trifluoride (Et_2_NSF_3_, abbreviated as DAST) was demonstrated. DAST is a commercially available liquid reagent that is utilized for the conversion of alcohols and carbonyl compounds into the corresponding fluoro derivatives. In spite of the effectiveness and convenience for nucleophilic fluorination, DAST is volatile and quite moisture sensitive, and readily undergoes disproportionation to SF_4_ and explosive (Et_2_N)_2_SF_2_ when heating at over 90 °C [41–43]. Therefore, special care is required in handling a large quantity of DAST concerning the danger of the explosion under heating conditions. To solve these technical issues, the use of DAST in a continuous-flow reactor employing inert plastic flow tubes provided flexibility, scale-up production, and improved safety of operation. Using flow chemistry, the conversion of alcohols to fluorides was achieved by Seeberger and Ley, independently (Scheme 2) [44–47]. Usually, *gem*-difluorination of ketones by DAST is known to be less efficient because it needs heating and/or long reaction time to complete the fluorination [48–49]. Nevertheless by the use of a flow microreactor device, isatin underwent *gem*-difluorination to give the corresponding difluoride in 73% yield (residence time in the reactor: within 1 h).

### Reactions that can be well-controlled and accelerated using flow microreactor systems

**Scheme 3:**
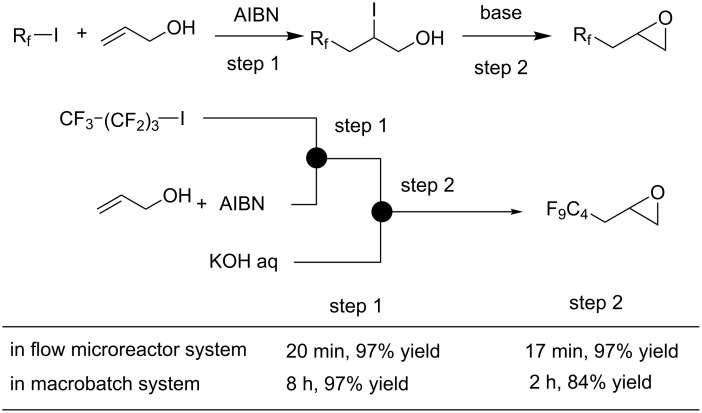
Flow microreactor synthesis of fluorinated epoxides.

The success of continuous flow chemistry in organic synthesis has enlarged rapidly last decade. There have been numerous examples using flow microreactors with improvement of chemical conversions and selectivities compared to conventional batch equipments. There are several beneficial effects using of flow microreactor systems; for instance, extremely fast mixing ability attributed to shortened diffusion path length, and highly efficient heat transfer ability based on large surface-area-to-volume ratio. In some cases, product selectively can be enhanced dramatically by the use of continuous flow microreactor devices. Daikin Industries, Ltd. developed the flow microreactor synthesis of fluorinated epoxides. In the first step (radical addition of perfluoroalkyl iodides to unsaturated alcohols), there is a problem concerning the violent exothermic reaction induced by decomposition of AIBN [50]. Furthermore, in the second step (intramolecular nucleophilic substitution of β-iodoalcohols to afford epoxides), efficient mixing technique is required for biphasic aqueous–organic systems. To resolve all the troublesome issues, flow microreactor provided improved reaction control over traditional batch reactors; high-yield synthesis of fluorinated epoxides was achieved (Scheme 3).

**Scheme 4:**
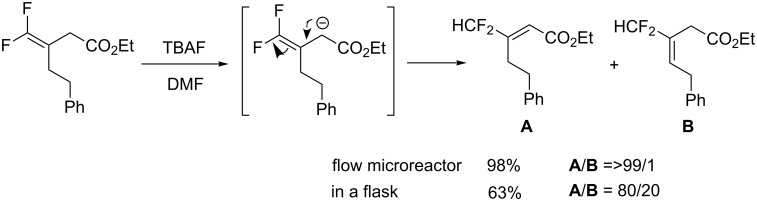
Highly controlled isomerization of *gem*-difluoroalkenes.

Kitazume et al. demonstrated the benefit of flow microreactors for a highly stereoselective synthesis of difluoromethylated alkenes [51]. They succeeded in perfect control of rapid base-catalyzed isomerization to give (*E*)-ethyl 3-difluoromethylpropenates (Scheme 4). Due to the benefit of temperature control in flow microreactor, further isomerization affording **B** was restricted.

**Scheme 5:**
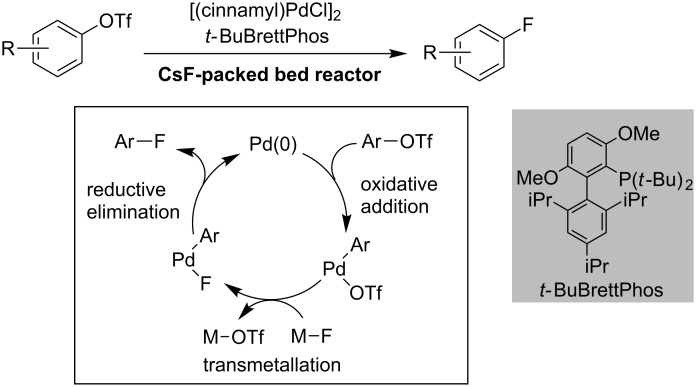
Flow system for catalytic aromatic fluorination.

The Pd-catalyzed fluorination of aryl triflates with fluoride was developed by Buchwald and co-workers in 2009 [52]. Both electron-rich and -poor aryl triflates underwent Pd-catalyzed aromatic fluorination. As a fluoride source, they used CsF, which is an expensive reagent. They found that the use of a large excesses of CsF shortened reaction times for catalytic aromatic fluorination. Furthermore, efficient mixing becomes difficult when dealing with large quantities of insoluble CsF. They invented a packed-bed flow reactor, which allowed for easy handling of large quantities of insoluble CsF with efficient mixing (Scheme 5) [53].

**Scheme 6:**
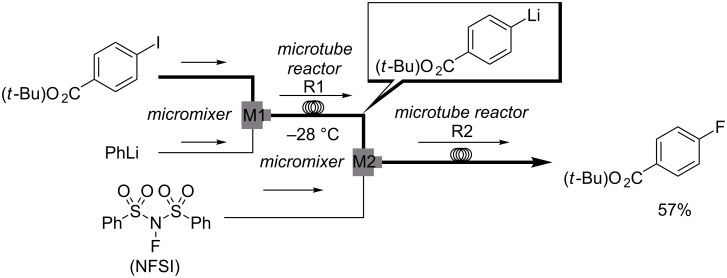
Continuous-flow reactor for electrophilic aromatic fluorination.

As an alternative method for aromatic fluorination, Yoshida and Nagaki reported the reactions of aryllithiums with electrophilic fluorinating agents such as NFSI and *N*-fluorosultam (Scheme 6) [54]. The present flow microreactor method showed a high level of functional group compatibility; a wide repertoire of aryl fluorides possessing electron-withdrawing, electron-donating, and sterically hindered functional groups were obtained in good yields. This is an important feature of 'late-stage fluorination methodology' for efficient drug screening.

**Scheme 7:**
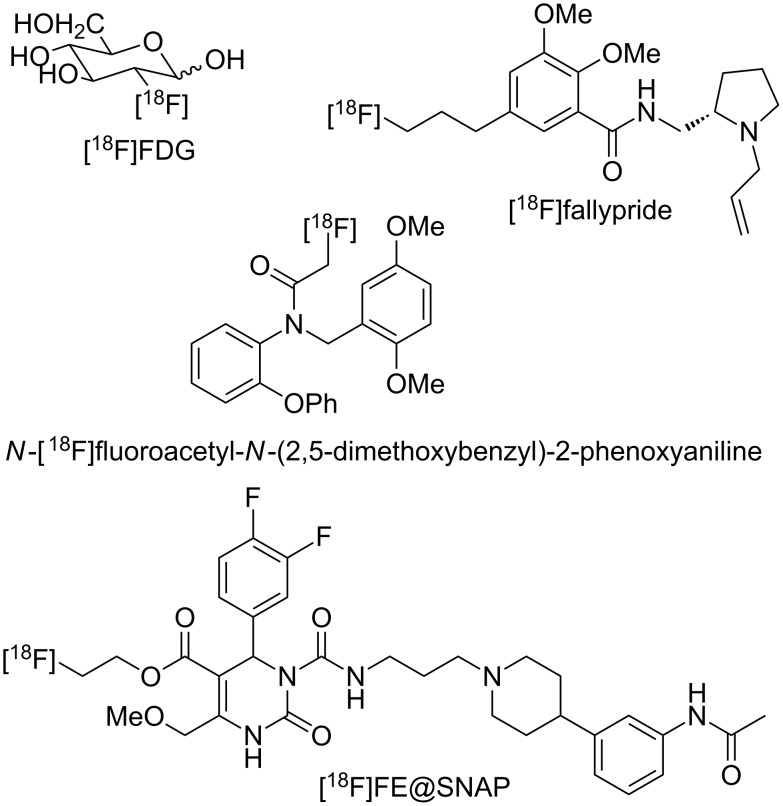
Examples of [^18^F]-radiolabeled molecular imaging probes.

[^18^F] is a fluorine radioisotope which acts as an important source of positrons. In the medical imaging modality, [^18^F]FDG is radiopharmaceutically used for positron emission tomography (PET). However, due to the short radioactivity elimination half-life of [^18^F] (*t*_1/2_ = 110 min), protocols for rapid and selective synthesis of [^18^F]-labeled compounds have been required. In addition, on-site and on-demand chemical production of PET agents with automation and ease of in-line purification is suitable for hospital use. To date, continuous flow microreactor technology has shown potential for synthesis of [^18^F]-radiolabeled molecular imaging probes such as [^18^F]FDG, [^18^F]fallypride, [^18^F]annexin, and so on, which were made by nucleophilic substitution reactions using [^18^F]-fluoride ion (Scheme 7) [55–67].

**Scheme 8:**
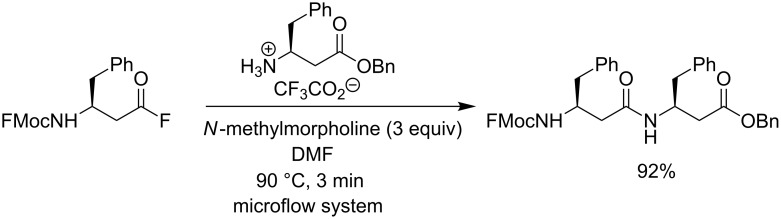
Flow microreactor synthesis of dipeptides.

For not only fluorination/fluoroalkylation, but also for defluorination, flow microreactor synthesis is quite effective. Defluorination involving a nucleophilic addition–elimination mechanism is a fundamental reaction in organic synthesis. Acid fluorides are known to be more stable to hydrolysis than acid chlorides under acidic or neutral conditions; on the other hand, they react with nucleophiles vigorously under basic conditions. Amino acid fluorides serve as one of the most efficient reagents for peptide bond formation. Seeberger and co-workers demonstrated the use of a silicon-based microreactor for the effective synthesis of peptides (Scheme 8) [68]. The condensation reaction completed in only 3 min at 90 °C to afford the dipeptide in high yield. Under the conditions at higher temperatures and/or leaving in prolonged contact with the reagents, decreasing the chemical yield of the desired dipeptide and increasing the amount of the tripeptide were observed. Using a flow microreactor system, precise residence-time control avoided the formation of undesired tripeptide byproduct.

**Scheme 9:**
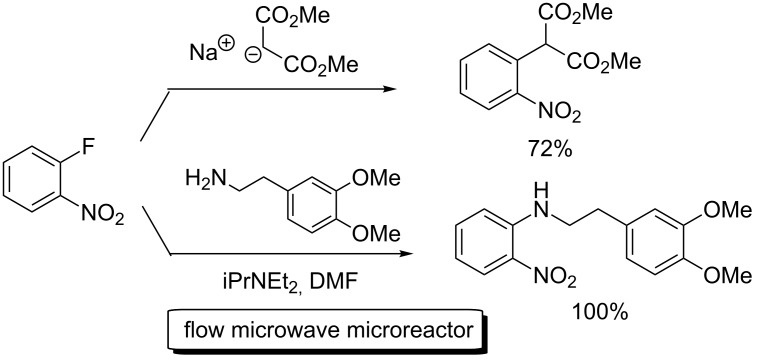
Flow synthesis involving S_N_Ar reactions.

**Scheme 10:**
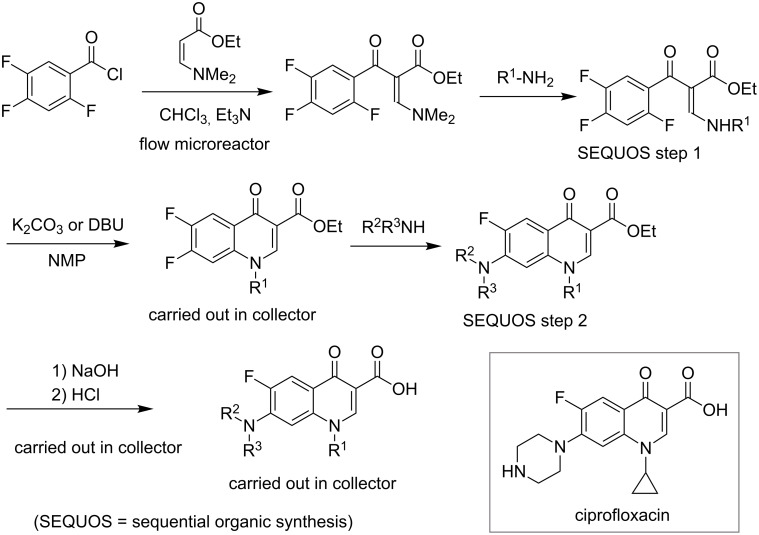
Flow synthesis of fluoroquinolone antibiotics.

Nucleophilic aromatic substitution (S_N_Ar) chemistry contributes to creating useful materials. In 2005, Comer and Organ reported S_N_Ar reactions of 2-fluoronitrobenzene using a flow microreactor system with microwave irradiation (Scheme 9) [69]. Toward making compound-libraries, Schwalbe explored a flow microreactor system for sequential transformation towards fluoroquinolone antibiotics such as ciprofloxacin via both inter- and intramolecular S_N_Ar reactions (Scheme 10) [70]. Starting from the acylation reaction of (*N*-dimethylamino)acrylate with 2,4,5-trifluorobenzoic acid chloride, followed by two kinds of library diversification involving 1) Michael addition of a set of primary amines and the subsequent nucleophilic ring closure to give the difluoroquinolone system, 2) intermolecular nucleophilic aromatic substitution with various amines and hydrolysis of ester moieties to afford a number of ciprofloxacin analogues in good overall yields with high purity.

### Reactions in which a reactive intermediate easily decomposes in batch reactors

As aforementioned, the benefits of flow microreactor synthesis are 1) accurate control of reaction time, temperature, and pressure, 2) efficient mixing, 3) improved product selectivity (yield and purity), 4) rapid removal of heat of reaction for increased process safety, 5) ease of scale-up from lab to plant scale, and so on.

The reaction times are governed by the residence times (concerning path lengths and flow rates) inside flow microreactor which allow the generation and reactions of reactive intermediates before decomposition. By virtue of these characteristic features, the residence time control in flow microreactors makes the generation of highly reactive species possible, including their reactions on a preparative scale within a second or less.

**Scheme 11:**
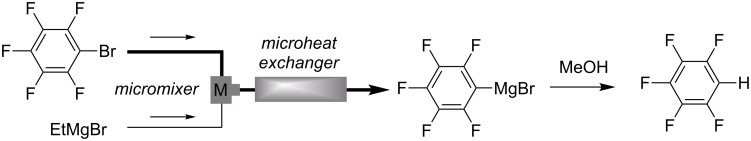
Highly controlled formation of PFPMgBr.

Pentafluorophenylmagnesium bromide (PFPMgBr) is industrially produced by means of the halogen–metal exchange reaction of ethylmagnesium bromide and bromopentafluorobenzene [71]. However, this process is a highly exothermic reaction, and therefore, the use of a slow addition technique is required to avoid rapid increase in temperature. The flow reactor systems with micromixer and microheat exchangers show efficacy of forming PFPMgBr (Scheme 11) [72]. In the well-controlled formation of PFPMgBr and the subsequent protonation, continuous running of the pilot plant-scale flow microreactor gave pentafluorobenzene (PFB) in 92% yield (14.7 kg).

**Scheme 12:**
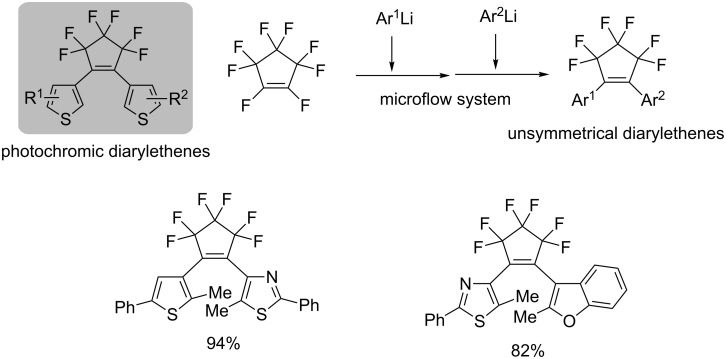
Selective flow synthesis of photochromic diarylethenes.

Molecular switches based on photochromic diarylethenes are one of the most promising electronic materials [73]. In particular, a number of 1,2-bis(heteroaryl)-substituted perfluorocyclopentenes have been synthesized for use as photosensitive components of photochromic recording media for optical memory. For the synthesis of 1,2-diarylperfluorocyclopentenes, octafluorocyclopentene undergoes very quick reaction with nucleophiles because of the extremely high electrophilicity of the double bond [74]. The nucleophilic substitutions take place in a stepwise manner via an addition–elimination pathway. However, it is difficult to synthesize unsymmetrical diarylethenes with conventional macro batch systems because of contamination of the symmetrical diarylethenes (two identical nucleophiles are incorporated at the same time). Integrated flow microreactor synthesis of photochromic diarylethenes was found to be effective; the generation of heteroaryllithiums and the subsequent nucleophilic addition/elimination with octafluorocyclopentene were successfully achieved (Scheme 12) [75]. As a significant progress, the selective synthesis of unsymmetrical diarylethenes was accomplished by stepwise introduction of different heteroaryl nucleophiles into octafluorocyclopentene with highly controlled manner [76].

**Scheme 13:**
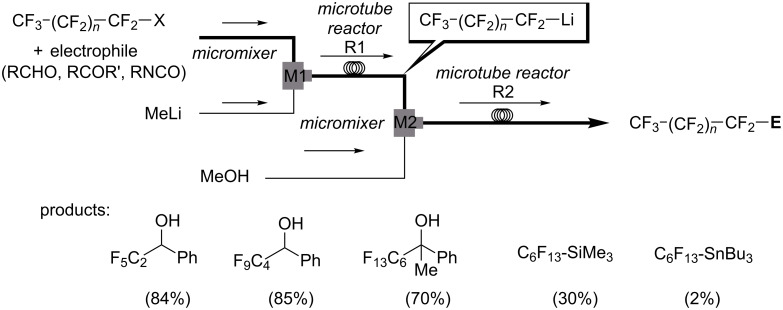
Flow microreactor system for perfluoroalkylation by generation of perfluoroalkyllithiums in the presence of electrophiles.

**Scheme 14:**
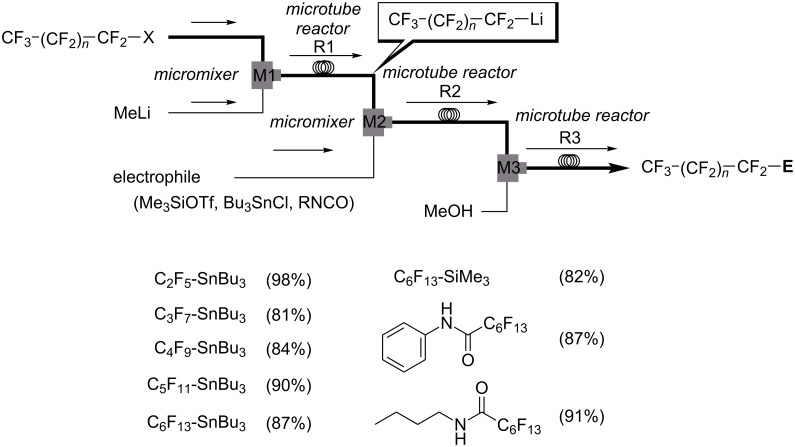
Integrated flow microreactor system for perfluoroalkylation by generation of perfluoroalkyllithiums in the absence of electrophiles.

The installation of perfluoroalkyl moieties into organic molecules can substantially changes the electronic properties of the parent organic compounds, and improves lipophilicity leading to enhanced solubility in fatty tissue and more efficient transport in the body. Nucleophilic introduction of perfluoroalkyl (R_F_) groups are one of the most effective and general methods for the synthesis of perfluoroalkylated compounds [77]. Perfluoroalkyllithiums, which are usually prepared by halogen–lithium exchange reactions of perfluoroalkyl halides with alkyllithiums are useful for nucleophilic perfluoroalkylation [78], however they readily undergo β-elimination to form perfluoroalkenes [79]. Flow microreactor systems were proved to be effective for generation and reactions of R_F_Li avoiding the β-elimination [80]. In the case of benzaldehyde as an electrophile, the reactions could be conducted at 0 °C, although much lower temperatures such as −78 °C are required to avoid the decomposition of perfluoroalkyllithium intermediates in batch processes (Scheme 13). However, the use of highly reactive electrophiles such as trimethylsilyl triflate and chlorotributylstannane gave rise to very low yields of the desired products, because the reactions of MeLi with such electrophiles are faster than those with perfluoroalkyl halides. In such cases, the generation of R_F_Li and the reactions with electrophiles should be separated to give the desired R_F_SiMe_3_ and R_F_SnBu_3_ in high yields. To solve the issue, the use of the integrated flow microreactor system was effective; well-controlled generation of highly reactive R_F_Li intermediates in the absence of active electrophiles and transfer of R_F_Li into the next step were accomplished by taking short residence times in the reactors before they decomposed (Scheme 14).

## Conclusion

The illustrious achievements have been made in developing organic reactions by the use of flow microreactor devices. Fluorinated organic compounds have been gaining a significant importance in wide field of science and technology, and there have been the associated growing need for new and more efficient synthetic methods. As highlighted here, each of these elegant flow microreactor syntheses surely supplies the innovative methods for the synthesis of organo-fluorine compounds. For the future, continuous flow microreactor chemistry will make a breakthrough in developing new chemical production with highly efficiency and unexploited fluorinated intermediates by the ingenious strategy, and will afford fluorine-containing useful materials.
